# Inferior Turbinate Size and CPAP Titration Based Treatment Pressures: No Association Found among Patients Who Have Not Had Nasal Surgery

**DOI:** 10.1155/2016/5951273

**Published:** 2016-01-19

**Authors:** Macario Camacho, Soroush Zaghi, Daniel Tran, Sungjin A. Song, Edward T. Chang, Victor Certal

**Affiliations:** ^1^Otolaryngology-Head and Neck Surgery, Division of Sleep Surgery and Medicine, Tripler Army Medical Center, 1 Jarrett White Road, Honolulu, HI 96859, USA; ^2^Department of Psychiatry and Behavioral Sciences, Sleep Medicine Division, Stanford Hospital and Clinics, Stanford, CA 94304, USA; ^3^Otolaryngology-Head and Neck Surgery, Division of Sleep Surgery and Medicine, Stanford Hospitals and Clinics, Stanford, CA 94304, USA; ^4^Department of Otorhinolaryngology, Sleep Medicine Centre, Hospital CUF, 4100-180 Porto, Portugal; ^5^Centre for Research in Health Technologies and Information Systems (CINTESIS), University of Porto, 4200-450 Porto, Portugal

## Abstract

*Objective*. To evaluate the effect of turbinate sizes on the titrated continuous positive airway pressure (CPAP) therapeutic treatment pressures for patients with obstructive sleep apnea (OSA) who have not had nasal surgery.* Study Design*. Retrospective case series.* Methods*. A chart review was performed for 250 consecutive patients.* Results*. 45 patients met inclusion criteria. The mean ± standard deviation (M ± SD) for age was 54.6 ± 22.4 years and for body mass index was 28.5 ± 5.9 kg/m^2^. The Spearman's rank correlation coefficient (*r*_*s*_) between CPAP therapeutic treatment pressures and several variables were calculated and were weakly correlated (age *r*_*s*_ = 0.29, nasal obstruction *r*_*s*_ = −0.30), moderately correlated (body mass index *r*_*s*_ = 0.42 and lowest oxygen saturation *r*_*s*_ = −0.47), or strongly correlated (apnea-hypopnea index *r*_*s*_ = 0.60 and oxygen desaturation index (*r*_*s*_ = 0.62)). No statistical significance was found with one-way analysis of variance (ANOVA) between CPAP therapeutic treatment pressures and inferior turbinate size (right turbinates *p* value = 0.2012, left turbinate *p* value = 0.3064), nasal septal deviation (*p* value = 0.4979), or mask type (*p* value = 0.5136).* Conclusion*. In this study, CPAP titration based therapeutic treatment pressures were not found to be associated with inferior turbinate sizes; however, the CPAP therapeutic treatment pressures were strongly correlated with apnea-hypopnea index and oxygen desaturation index.

## 1. Introduction

There are several medical [1] and surgical [2, 3] treatment options for obstructive sleep apnea (OSA). Patients who use continuous positive airway pressure (CPAP) devices have been shown to have nasal obstruction as a common complaint (estimated prevalence: 25–45%) [4–6]. As described by Poiseuille's Law, airflow resistance is proportional to the length and is inversely proportion to the radius to the fourth power [7]. Because the radius is such an important variable, small changes, such as a 10% increase in the cross-sectional area of the nasal cavity airway, can result in a 21% increase in airflow [8]. Although surgery on the nose has not been shown to dramatically improve OSA [9], it can improve CPAP device use [10].

A recent systematic review and meta-analysis also demonstrated that isolated nasal surgery reduces CPAP device therapeutic treatment pressures by 2-3 centimeters of water pressure (cwp) [10]. Therefore, surgically increasing the size of the nasal airway decreases nasal resistance and reduces CPAP device pressure requirements [10]. However, to our knowledge, for patients who have not undergone nasal surgery it is unknown whether patients with smaller turbinates have lower CPAP therapeutic treatment pressure requirements when compared to patients with larger turbinates. A recently published systematic review did not identify any study in the international literature that used inferior turbinate size as a variable in mathematical equations to predict CPAP [11]. For this study we hypothesized that, in patients who have not had nasal surgery, large turbinates would require higher CPAP therapeutic treatment pressures than small inferior turbinates. Because it has previously been shown that nasal surgery can reduce CPAP therapeutic treatment pressures [12], we planned to exclude patients with prior nasal surgery in order to remove this confounding variable. The objective of this study is to evaluate the effect of turbinate sizes on the CPAP titration based therapeutic treatment pressures (in centimeters of water pressure) for patients with OSA who have not previously undergone nasal surgery.

## 2. Materials and Methods

The Stanford Hospital and Clinics Institutional Review Board was contacted and written approval was granted prior to commencing this study. The study design is a retrospective case series evaluating 250 consecutive patients. Inclusion criteria are as follows: (1) Stanford Sleep Medicine Clinic patients who had a nasal examination and underwent an attended in-lab CPAP titration study and (2) the nasal examination needed to include nasal septal deviation severity and inferior turbinate grades for the left and right sides separately. Exclusion criteria are as follows: (1) patients who have undergone nasal surgery. The CPAP titration pressures were obtained based on overnight, in-lab polysomnography. The American Academy of Sleep Medicine (AASM) Manual for the Scoring of Sleep and Associated Events was used by Stanford and outside institutions. The Stanford hypopnea scoring criteria included ≥10 seconds with ≥30% reduction in airflow measured by the nasal flow transducer associated with a 3% desaturation and/or an electroencephalogram arousal as described in the AASM scoring manual 2013, version 2.0.2 [13].

**Figure 1:**
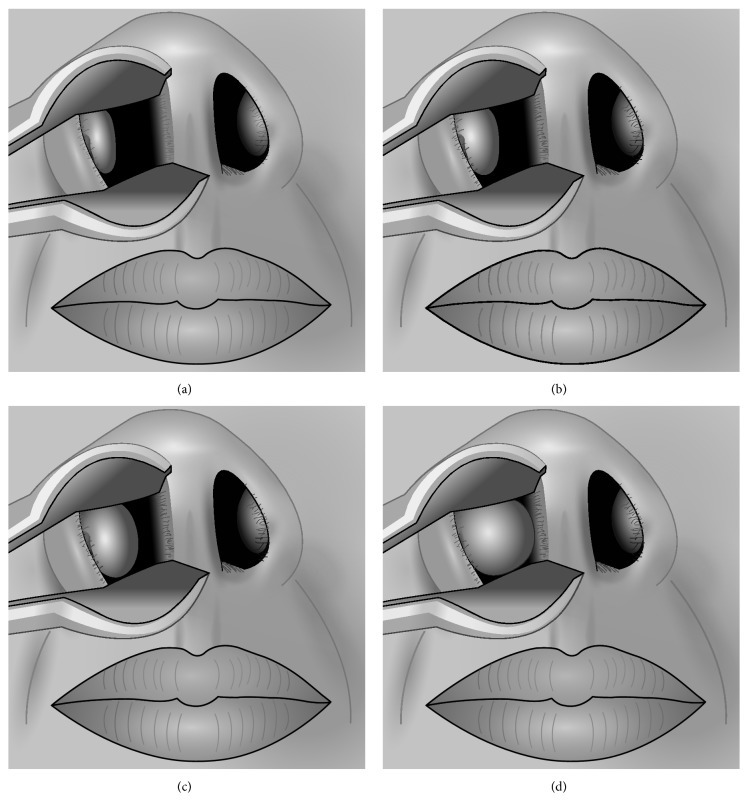
(a) Grade 1 (0%–25% of total airway space). (b) Grade 2 (26%–50% of total airway space). (c) Grade 3 (51%–75% of total airway space). (d) Grade 4 (76%–100% of total airway space). Reproduced with permission [12].

In order to fully evaluate the effect of inferior turbinate size, a tool (“Inferior Turbinate Classification System, Grades 1 to 4”) [12] was utilized. This Inferior Turbinate Classification System provides a method for grading the amount of airway space that the anterior aspect of the inferior turbinate occupies relative to the total available airway space and is summarized as follows: grade 1 is 0–25% of the total airway space, grade 2 is 26–50% of the total airway space, grade 3 is 51–75% of the total airway space, and grade 4 is 76–100% of the total airway space [12]; see Figure 1. The Nasal Obstruction Symptom Evaluation (NOSE) scale was used to evaluate nasal obstruction and a patient with a score >40 was considered to have nasal obstruction [14].

## 3. Statistical Analysis

The data was cataloged using Microsoft Excel 2013 (Redmond, WA, USA). The IBM Statistical Package for Social Sciences (SPSS) software version 20 (Armonk, New York, USA) was used for statistical analyses. The patient data was analyzed by calculating the means, standard deviations (M ± SD), and 95% confidence intervals [95% CI]. One-way analysis of variance (ANOVA) was used to evaluate ordinal and nominal data; Spearman's rank correlation coefficient (*r*_*s*_) was used for continuous data measures. The *r*_*s*_ was selected for correlating variables because it is less sensitive to strong outliers and it can also be used for calculating correlation coefficients for both continuous and discrete variables. The standard recommendations for *r*_*s*_ strengths were used [15]: 0.0–0.19 = very weak, 0.20–0.39 = weak, 0.40–0.59 = moderate, 0.60–0.79 = strong, and 0.80–1.0 = very strong. Variables evaluated included the CPAP titration data, age, and body mass index (BMI) in kilograms per meter squared (kg/m^2^), race/ethnicity, apnea-hypopnea index (AHI), oxygen desaturation index (ODI), lowest oxygen saturation (LSAT), inferior turbinate size, nasal septal deviation severity, and other physical exam findings. For CPAP titration pressures, if a fixed pressure was prescribed, that value was used and if pressure ranges were prescribed, then the average of the pressure range was calculated and used as the CPAP therapeutic treatment pressure for analysis purposes. Multivariate analysis was performed using Standard Least Squares Linear Regression. A two-tailed *p* value < 0.05 was considered statistically significant.

## 4. Results

**Figure 2:**
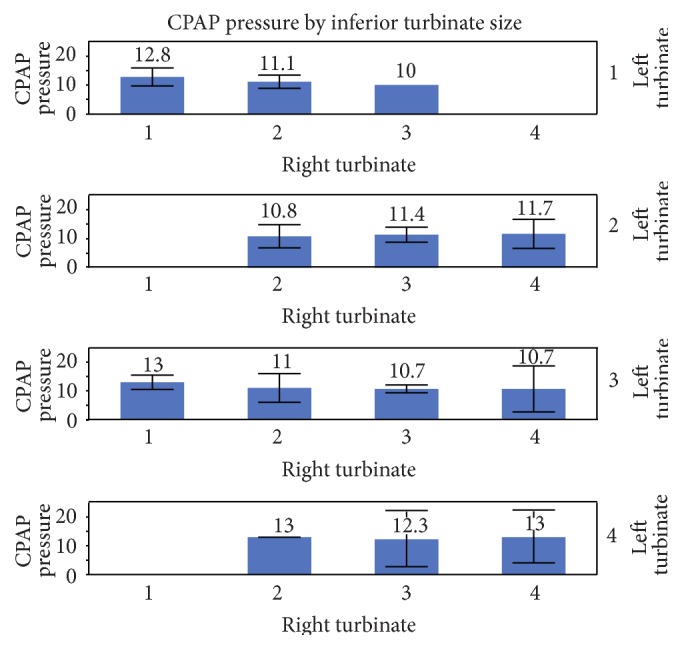
Mean diagnostic CPAP by inferior turbinate size (grades 1–4). Each error bar is constructed using a 95% confidence interval of the mean. Multivariate analysis with Standard Least Squares Linear Regression Model shows *R* ^2^ = 0.08, *p* value = 0.9953 consistent with no association to very weak association between CPAP and inferior turbinate size.

A total of 45 patients met study inclusion criteria. The mean ± standard deviation (M ± SD) for age was 54.6 ± 22.4 years and for body mass index was 28.5 ± 5.9 kg/m^2^.  Table 1 provides demographic information for the patients to include age, AHI, BMI, ODI, LSAT, NOSE Scale scores, race information, nasal septal deviation severity, inferior turbinate size, and mask type. The Spearman's rank correlation coefficient (*r*_*s*_) between CPAP therapeutic treatment pressures and several variables were calculated and were weakly correlated (age *r*_*s*_ = 0.29, nasal obstruction *r*_*s*_ = −0.30), moderately correlated (body mass index *r*_*s*_ = 0.42 and lowest oxygen saturation *r*_*s*_ = −0.47), or strongly correlated (apnea-hypopnea index *r*_*s*_ = 0.60 and oxygen desaturation index (*r*_*s*_ = 0.62)). No statistical significance was found with one-way analysis of variance (ANOVA) between CPAP therapeutic treatment pressures and inferior turbinate size (right turbinates *p* value = 0.2012, left turbinate *p* value = 0.3064), nasal septal deviation (*p* value = 0.4979), or mask type (*p* value = 0.5136); see Table 2. The M ± SD for therapeutic CPAP for grade 1 (five patients): 12.8 ± 2.5 cwp, grades >1 to 2 (eleven patients): 11.5 ± 1.6 cwp, grades >2 to 3 (twenty-one patients): 11.3 ± 1.8 cwp, and grades >3 to 4 (eight patients): 12.2 ± 2.9 cwp, with a one-way ANOVA *p* value of 0.4599; see Table 3. Mean diagnostic CPAP titration based treatment pressure by inferior turbinate size (grades 1–4) was evaluated with multivariate analysis with the Standard Least Squares Linear Regression Model with an *R* ^2^ = 0.08, *p* value = 0.9953 consistent with no association to very weak association; see Figure 2.

### 4.1. Sub-Analyses

#### 4.1.1. Nasal Obstruction versus No Nasal Obstruction

Among a subgroup analysis of patients without nasal obstruction (as evaluated by a NOSE Scale Score [14] of 40 out of 100 or less, *n* = 34 patients) the M ± SD for age was 56.0 ± 23.8 years, for body mass index was 28.4 ± 6.6 kg/m^2^, and for inferior turbinate size was 2.47 ± 0.80. The M ± SD for CPAP therapeutic treatment pressures for all 34 patients was 11.8 ± 2.2 cwp, for grade 1 (four patients): 13.3 ± 2.7 cwp, grades >1 to 2 (seven patients): 11.6 ± 1.7 cwp, grades >2 to 3 (eighteen): 11.5 ± 1.8 cwp, and grades >3 to 4 (five patients) 12.1 ± 3.5 cwp, with a one-way ANOVA *p* value of 0.5213; see Table 3. For patients with complaints of nasal obstruction (11 patients) the M ± SD for age was 62.5 ± 15.4 years, for body mass index was 29.2 ± 3.5 kg/m^2^, and for inferior turbinate size was 2.3 ± 0.9. The M ± SD for CPAP therapeutic treatment pressures for all 11 patients was 11.1 ± 1.3 cwp, for grade 1 (one patient): 11 cwp, grades >1 to 2 (four patients): 11.3 ± 1.5 cwp, grades >2 to 3 (three patients): 10.0 ± 0.0 cwp, and grades >3 to 4 (three patients) 12.5 ± 0.7 cwp, with a one-way ANOVA *p* value of 0.4722; see Table 3.

#### 4.1.2. Nasal Mask Type

There were three categories in the subanalysis for mask type: unknown mask types (7 patients), nasal masks (27 patients), and oronasal masks (11 patients). The M ± SD for CPAP therapeutic treatment pressures for the seven patients with an unknown mask type was 11.3 ± 1.7 cwp; the M ± SD turbinate sizes were 2.66 ± 0.30. The M ± SD for CPAP therapeutic treatment pressures for twenty-seven patients with nasal masks was 11.8 ± 2.3 cwp; the M ± SD turbinate sizes were 2.41 ± 0.96. For nasal masks, the one-way ANOVA *p* value of 0.9217, see Table 3. The M ± SD for CPAP therapeutic treatment pressures for eleven patients with oronasal masks was 11.2 ± 1.5 cwp; the M ± SD for turbinate sizes was 2.45 ± 0.82. For oronasal masks, the one-way ANOVA *p* value of 0.2732, see Table 3.

## 5. Discussion

There are two main findings to this study. First, CPAP therapeutic treatment pressures do not seem to be influenced by inferior turbinate sizes in patients who have not undergone nasal surgery. It has been shown that patients who have undergone nasal surgery will have a decrease in CPAP therapeutic treatment pressures by approximately 2-3 centimeters of water pressure [10]; therefore, patients with prior nasal surgery were intentionally excluded from the study in order to eliminate this variable as a confounder. The mean diagnostic CPAP and inferior turbinate sizes (grades 1–4) were evaluated with multivariate analysis with the Standard Least Squares Linear Regression Model with an *R* ^2^ = 0.08, *p* value = 0.9953 consistent with no association to very weak association.

Second, other variables were better correlated with CPAP therapeutic treatment pressures. There was a weak correlation between CPAP therapeutic treatment pressures and nasal obstruction using the NOSE Scale questionnaire (*r*_*s*_ was −0.21, two-tailed *p* value 0.57, not statistically significant) and a very weak correlation for patients without nasal obstruction using the NOSE Scale questionnaire (*r*_*s*_ was −0.05, two-tailed *p* value 0.78, not statistically significant). Given the lack of an association of the inferior turbinate sizes, nasal septal deviation severity, and nasal obstruction overall, these findings suggest that simply observing nasal abnormalities in a patient may not warrant surgery if they do not have complaints of nasal obstruction. Another finding was that lowest oxygen saturation *r*_*s*_ = −0.47 and body mass index (*r*_*s*_ = 0.42) were moderately correlated. The moderate correlation with BMI is not unexpected as it is logical that a larger person would require more pressure than a thin person given the additional mass in the upper airway and in the abdomen. Two variables, apnea-hypopnea index (*r*_*s*_ = 0.60) and oxygen desaturation index (*r*_*s*_ = 0.62), were strongly correlated, which is a logical finding given that a CPAP titration is intended to reduce arousals and to improve oxygenation.

Additional research is needed in order to evaluate whether CPAP therapeutic treatment pressures are truly independent of inferior turbinate sizes. As a retrospective case series utilizing chart review, this study provides level 4 evidence. We would like to encourage researchers to incorporate and use the “Inferior Turbinate Classification System, Grades 1–4” as it is a tool which has high intra- and interrater reliability. By using the classification system, the influence that the inferior turbinate sizes have as related to nasal obstruction and CPAP can be more accurately ascertained. Furthermore, despite the lack of an association between CPAP therapeutic treatment pressures and inferior turbinate sizes in patients without nasal surgery we would still recommend that patients with nasal obstruction and large turbinates undergo turbinoplasties as several studies have demonstrated a quality of life benefit and improvement in CPAP use and decreased CPAP in patients who have undergone nasal surgery [10]. Additionally, to our knowledge, this study is the first to evaluate the association between inferior turbinate sizes and therapeutic CPAP; therefore, we caution against making generalizations. In order to increase the level of evidence, we would also encourage prospective case series, case-control, cohort, and even randomized controlled trials. Once several studies have been published, a systematic review and meta-analysis would more accurately answer the question using statistical analysis with random effects modeling.

## 6. Limitations

There are limitations to this study. First, we are limited to the constraints which are shared by all retrospective studies in that only the previously collected data could be utilized and analyzed; therefore, if there are missing data, then patients may have had to be excluded solely based on the lack of documentation. Second, in this study we did not review CPAP device pressures for patients who had previous nasal surgery; however, this was done intentionally given that a meta-analysis of eighteen studies demonstrated a reduction by 2-3 centimeters of water pressure after isolated nasal surgeries [10]. Third, given that we did not have rhinomanometry nor acoustic rhinometry, we were not able to evaluate the relationship between the data from these tools and the inferior turbinate sizes and nasal function as it relates to CPAP; future studies could evaluate these relationships. Lastly, there was no rigid or flexible endoscopy performed in the assessment of these patients as the sleep medicine clinics do not have them available; however, each patient underwent a nasal examination by the first author who is a board-certified otolaryngologist.

## 7. Conclusion

In this study, CPAP titration based therapeutic treatment pressures were not found to be associated with inferior turbinate sizes; however, the CPAP therapeutic treatment pressures were strongly correlated with apnea-hypopnea index and oxygen desaturation index.

## Figures and Tables

**Table 1:** Variables for the patients included in the study.

Variables	*N*	M ± SD
All patients		
Age (years)	45	54.6 ± 22.4
AHI (events/hr)	44	34.7 ± 29.4
BMI (kg/m^2^)	45	28.5 ± 5.9
ODI (events/hr)	20	27.3 ± 32.7
LSAT (percent)	42	85.9 ± 6.2
NOSE Score (scaled 0–100)	45	28.9 ± 22.5
		*CPAP *
All patients	45	11.6 ± 2.0 cwp
Asian	6	12.0 ± 1.4 cwp
Black	3	13.3 ± 3.5 cwp
Caucasian	29	11.6 ± 2.0 cwp
Indian	4	11.4 ± 2.1 cwp
Latino	3	9.8 ± 0.8 cwp
Nasal deviation severity	40	
Grade 1 (0–25%)	24	12.0 ± 2.3 cwp
Grade 2 (26–50%)	11	11.0 ± 1.6 cwp
Grade 3 (51–75%)	3	10.8 ± 1.0 cwp
Grade 4 (76–100%)	2	11.0 ± 1.4 cwp
Inferior turbinate size: right		
Grade 1 (0–25%)	8	12.9 ± 2.0 cwp
Grade 2 (26–50%)	12	11.3 ± 1.5 cwp
Grade 3 (51–75%)	16	11.1 ± 1.7 cwp
Grade 4 (76–100%)	9	11.8 ± 2.8 cwp
Inferior turbinate size: left		
Grade 1 (0–25%)	10	11.9 ± 2.1 cwp
Grade 2 (26–50%)	11	11.3 ± 1.8 cwp
Grade 3 (51–75%)	17	11.1 ± 2.0 cwp
Grade 4 (76–100%)	7	12.8 ± 2.2 cwp
Mask type		
Unknown	7	11.3 ± 1.7 cwp
Nasal mask	27	11.8 ± 2.3 cwp
Oronasal mask	11	11.2 ± 1.5 cwp

AHI = apnea-hypopnea index; CPAP = continuous positive airway pressure; LSAT = lowest oxygen saturation; *N* = number of patients in the study with data available; NOSE Score = Nasal Obstruction Symptom Evaluation Scale score; and ODI = oxygen desaturation index.

**Table 2:** Results of statistical tests for patient variables versus CPAP prescription pressures. Spearman's rank correlation coefficient (*r*_*s*_) was used for continuous data measures; one-way ANOVA was used to evaluate ordinal and nominal data. A multivariate model was developed with Standard Least Squares Linear Regression using factors identified as significant on univariate analysis: AHI, BMI, LSAT, NOSE, and ODI (*n* = 21 observations, *R* ^2^ = 0.56, *p* = 0.0030); parameter estimates and standard errors are shown.

Variables versus CPAP	*N*	Correlation coefficient	Univariate statistical test, *p* value	Multivariate analysis: Standard Least Squares Linear Regression Model		
		*r*_*s*_	Spearman's rank correlation	β estimate	Standard error	*p* value
Age (years)	45	0.29	0.058	Not included		
AHI (events/hr)	44	0.60	0.000005^*∗*^	0.10	0.04	0.0131^*∗*^
BMI (kg/m^2^)	45	0.42	0.0036^*∗*^	0.13	0.05	0.0233^*∗*^
LSAT (percent)	42	−0.47	0.0017^*∗*^	−0.05	0.06	0.3804
NOSE Score (scaled 0–100)	45	−0.30	0.048^*∗*^	−0.006	0.018	0.7449
ODI (events/hr)	20	0.62	0.0038^*∗*^	−0.085279	0.036548	0.0340^*∗*^
			*One-way ANOVA*			
Inferior turbinate size (1–4)	45	N/A	Right turbinate: 0.2012	Not included		
			Left turbinate: 0.3064	Not included		
Nasal septal deviation (1–4)	40	N/A	0.4979	Not included		
Mask type: nasal versus oronasal	38	N/A	0.5136	Not included		

^*∗*^Statistically significant (*p* value < 0.05). AHI = apnea-hypopnea index; BMI = body mass index; CPAP = continuous positive airway pressure; LSAT = lowest oxygen saturation; NOSE Score = Nasal Obstruction Symptom Evaluation Scale score; and ODI = oxygen desaturation index.

**Table 3:** Therapeutic continuous positive airway pressures, body mass index, and NOSE scale scores stratified by median inferior turbinate sizes. *p* values from statistical testing with one-way ANOVA are shown.

Variables	Median inferior turbinate size: right and left				*p* value
	Grade 1	Grade >1 to 2	Grade >2 to 3	Grade >3 to 4	
All patients (*N* = 45)	*N* = 5	*N* = 11	*N* = 21	*N* = 8	
BMI	28.1 ± 4.4	29.1 ± 6.1	27.7 ± 4.9	30.6 ± 9.7	0.8222
CPAP	12.8 ± 2.5	11.5 ± 1.6	11.3 ± 1.8	12.2 ± 2.9	0.4599
NOSE Score	33.0 ± 25.1	30.0 ± 18.1	26.0 ± 25.7	30.4 ± 20.7	0.8602
Patients w/o nasal obstruction (*N* = 34)	*N* = 4	*N* = 7	*N* = 18	*N* = 5	
BMI	26.8 ± 3.9	28.8 ± 7.5	28.0 ± 5.2	30.3 ± 11.7	0.8744
CPAP	13.3 ± 2.7	11.6 ± 1.7	11.5 ± 1.8	12.1 ± 3.6	0.5213
NOSE Score	23.8 ± 16.5	18.9 ± 11.2	16.8 ± 12.1	19.5 ± 11.2	0.7766
Patients with nasal obstruction (*N* = 11)	*N* = 1	*N* = 4	*N* = 3	*N* = 3	
BMI	33.0	29.7 ± 3.3	25.6 ± 1.9	31.3 ± 3.2	0.2911
CPAP	11.0	11.3 ± 1.5	10.0 ± 0.0	12.5 ± 0.7	0.4722
NOSE Score	70	49.4 ± 7.7	80.8 ± 8.0	57.5 ± 3.5	0.1125
Patients using nasal mask (*N* = 27)	*N* = 4	*N* = 7	*N* = 12	*N* = 4	
BMI	29.1 ± 4.3	28.1 ± 5.8	27.9 ± 4.2	30.5 ± 13.5	0.9122
CPAP	12.3 ± 2.5	11.9 ± 1.5	11.4 ± 1.7	11.9 ± 4.1	0.9217
NOSE Score	31.3 ± 28.7	25.7 ± 16.3	22.7 ± 20.4	21.8 ± 11.4	0.8826
Patients using oronasal mask (*N* = 11)	*N* = 1	*N* = 4	*N* = 4	*N* = 2	
BMI	23.9	31.1 ± 7.0	23.2 ± 5.4	26.8 ± 3.2	0.3645
CPAP	15.0	10.8 ± 1.5	10.9 ± 2.2	11.0 ± 1.4	0.2732
NOSE Score	40	37.5 ± 21.0	33.8 ± 39	55.0 ± 7.1	0.8611

BMI = body mass index in kg/m^2^; CPAP = continuous positive airway pressure; *N* = number of patients; NOSE Score = Nasal Obstruction Symptom Evaluation questionnaire [14].
